# Improving the Shelf Life of Avocado Fruit against *Clonostachys rosea* with Chitosan Hybrid Films Containing Thyme Essential Oil

**DOI:** 10.3390/polym14102050

**Published:** 2022-05-17

**Authors:** Wendy Abril Coyotl-Pérez, Efraín Rubio-Rosas, Quetzali Nicte Morales-Rabanales, Sergio Alberto Ramírez-García, Yesenia Pacheco-Hernández, Victor Hugo Pérez-España, Omar Romero-Arenas, Nemesio Villa-Ruano

**Affiliations:** 1 Centro de Agroecología, Instituto de Ciencias, Benemérita Universidad Autónoma de Puebla, San Pedro Zacachimalpa, Puebla 72960, Mexico; wendy.coyotl@alumno.buap.mx; 2 Centro Universitario de Vinculación y Transferencia de Tecnología, Benemérita Universidad Autónoma de Puebla, Puebla 72570, Mexico; efrain.rubio@correo.buap.mx; 3 Unidad Profesional Interdisciplinaria Ingeniería Tecnología Avanzada, Instituto Politécnico Nacional, Av. Instituto Politécnico Nacional 2580, La Laguna Ticoman, Gustavo A. Madero, Ciudad de México 07340, Mexico; qmoralesr1800@alumno.ipn.mx; 4 Instituto de Nutrición, Universidad de la Sierra Sur, Guillermo Rojas Mijangos, Col. Ciudad Universitaria, Miahuatlán de Porfirio Díaz, Oaxaca 70800, Mexico; aramirez@unsis.edu.mx; 5 Centro de Investigación y de Estudios Avanzados del IPN, Unidad Irapuato, Km 9.6 Carretera Irapuato-León, Irapuato 36824, Mexico; yesenia.pacheco@cinvestav.mx; 6 Escuela Superior de Apan, Universidad Autónoma del Estado de Hidalgo, Carretera Apan-Calpulalpan Km 8, Chimalpa 43920, Mexico; victorhugo_perez@uaeh.edu.mx; 7 CONACyT-Centro Universitario de Vinculación y Transferencia de Tecnología, Benemérita Universidad Autónoma de Puebla, Puebla 72570, Mexico

**Keywords:** hybrid films, Hass avocadoes, thyme essential oil, *Clonostachys rosea*, nutraceuticals

## Abstract

Hass avocadoes are one of the most popular fruits consumed worldwide because of their nutritional and nutraceutical content. Nevertheless, these fruits are susceptible to phytopathogen attacks that decrease fruit quality during the postharvest period. Herein we present the results of the in situ fungistatic activity of four hybrid films (FT1–FT4) manufactured with chitosan and different concentrations of the essential oil of thyme (TvEO). The films were evaluated as biodegradable materials to prevent fruit decay triggered by *Clonostachys rosea* which is considered an emergent phytopathogen of this crop. The in situ fungistatic strength, spectroscopic properties (FT-IR), optical features (transmittance/opacity), and consistency obtained by microscopic analysis (SEM), indicated that the films FT3 and FT4 possessed the best physicochemical properties to protect Hass avocadoes against the soft rot produced by *C. rosea*. Avocadoes treated with the films FT3 and FT4 significantly (*p* < 0.01) conserved fruit firmness and nutritional composition (protein, fat, fiber, and reducing sugars) as well as the nutraceutical content (oleic, palmitoleic, linoleic, and palmitic acids) of infected avocados for 21 days. Our results validate the potential use of the films FT3 and FT4 to prevent the soft rot caused by *C. rosea* and to improve the shelf life of Hass avocadoes.

## 1. Introduction

Mexico is the main producer and exporter of Hass avocado (*Persea americana* Mill.) around the world [1]. This fruit is considered a valuable source of vitamins, fiber, phenols, and minerals which have an evident role in human physiology [2]. The fat content of the avocado fruit is highly appreciated by consumers because of the presence of unsaturated fatty acids which benefit human health [2]. Like other fruits, avocadoes are susceptible to phytopathogenic attacks because of their content of fat, protein, fiber, and reducing sugars which are nutrients for a broad spectrum of micro-organisms. As a consequence, fruit quality is substantially affected, and severe economic losses are produced during the postharvest period [3]. The postharvest diseases triggered by *Colletotrichum*, *Lasiodiplodia*, *Sphaceloma*, *Pseudocercospora*, *Rhizopus*, and *Fusarium* genera are the most devastating threats to avocado producers around the world [3].

Despite several fungal species being able to reduce avocado production, new organisms with phytopathogenic activity can be found under open field conditions [4]. The phytopathogenic behavior of these organisms is usually associated with specific climatic and soil features in which the avocado plants grow [5]. The state of Puebla is the sixth largest producer of avocado in Mexico and the northern highlands of this state are considered a new zone dedicated to the production of this fruit. Very recently, a wild strain of *Clonostachys rosea* (C08-9) was reported as a new and devastating phytopathogen for the avocadoes harvested in the northern highlands of Puebla-Mexico [2]. This phytopathogen produces an aggressive fruit blotch which led to severe economic losses.

Approximately 1.3 billion tons of fruits and vegetables are wasted around the world [6]. In developing countries, the percentage of vegetables that are wasted is near 80% [7]. Due to this fact, emerging alternatives, such as bio-based polymers obtained from renewable materials, represent a potential solution to avoid the excessive waste of food because of their capacity to extend shelf life [6]. In this context, biofilms made with chitosan and essential oils have attracted great interest in food packaging due to their biodegradability, low toxicity, and strong antimicrobial activity [8]. Due to this fact, this investigation aimed to evaluate the in situ fungistatic activity of hybrid films containing the essential oil from thyme (*Thymus vulgaris* L.) on *C. rosea* strain C08-9, which is considered an emergent threat to avocado producers. Also, we determined the effect of these films on the conservation of avocados’ fruit quality.

## 2. Materials and Methods

### 2.1. Plant Material and Assays with the Causal Agent of Avocado Rot

Diseased avocadoes were collected during June 2021 in Yaonáhuac Puebla, Mexico (19°56′55″ N 97°26′26″ W; 1997 masl) as reported by Coyotl-Pérez et al. [2].

The pathogenicity tests were carried out in asymptomatic Hass avocadoes (n = 20) collected in the zone of study in accordance with Pérez-Vázquez et al. [9]. Pathogenicity tests were done in accordance with Coyotl-Pérez et al. [2] using the strain C08-9 of *C. rosea* which was isolated from diseased avocadoes. This phytopathogen was previously identified using the methods described by Pérez-Vázquez et al. [10]. For this purpose, 1000 conidia were obtained from a monosporic culture (10 µL of a sterile saline solution containing 1 × 10^5^ conidia mL^−1^). The conidia were inoculated by mechanical penetration using a Hamilton syringe into the pericarp of sanitized asymptomatic avocadoes (n = 20). The samples were maintained at 28 °C and 70% relative humidity for 21 days. The control group (n = 20) was treated only with an equivalent volume of saline solution. The sequences of the internal transcribed spacer (ITS) of the 18S ribosomal gene and the translation elongation factor 1 alpha gene (TEF-1 alpha were stored in the nucleotide database of the National Center for Biotechnology Information (NCBI; Bethesda, MD, USA) with the accessions OM473286 and OM715999, respectively).

### 2.2. Thyme Essential Oil Extraction and Analysis

Dried leaves of *Thymus vulgaris* were purchased in the central market of Puebla City (19°05′21.6″ N and 98°11′14.3″ W) in June 2021. Ten kg of dried material were extracted by hydrodistillation with a Clevenger trap for 3 h. The essential oil of *T. vulgaris* (TvEO) was dissolved in *n*-hexane, dried under sodium sulfate, and stored at 4 °C until use. The chemical profile of TvEO was determined using the same equipment, capillary column, and analytical conditions previously described by Morales-Rabanales et al. [11].

### 2.3. In Vitro Antifungal Experiments with TvEO

Hyphal discs of *C. rosea* (3 mm diameter) were extracted from potato dextrose agar (PDA) cultures (10 d) using the nozzle connection of a sterile micropipette tip (200 µL capacity). The discs were deposited in 96-well plates containing 3 × 10^−4^ L potato dextrose broth (PDF; Bioxon^®^, Mexico City, Mexico) and mixed with 1 × 10^−3^ M resazurin sodium salt (Sigma-Aldrich Co., St. Louis, MO, USA) [12]. The minimum inhibitory concentration (MIC) was determined by dose–response curves of TvEO (1–600 mg L^−1^). Readings were recorded at 630 nm after incubation at 28 °C for 48 h. Each concentration was assayed twenty-five times (n = 25).

### 2.4. Preparation of Hybrid Films with TvEO and Chitosan

The films were prepared in accordance with Morales et al. [11]. Chitosan (1% *w*/*v*; medium molecular weight purchased from Sigma-Aldrich Co., St. Louis, MO, USA) was dissolved in 1% (*v*/*v*) acetic acid. Then, four concentrations of TvEO (0.4, 0.7, 1.0, and 1.3% *w*/*v*) were added to 0.1 L of the chitosan solution and mixed under constant stirring at 50 °C for 1 h. The resulting coatings were named FT1, FT2, FT3, and FT4. Twenty milliliters of each mixture were distributed in glass Petri dishes (90 mm diameter) and dried at room temperature (25 °C) for 72 h. The films (n = 25) were subjected to further physicochemical analysis and biological assessment. Pure chitosan films (Ch) without TvEO were used as controls of fungistatic activity. The thickness of the films was measured using a high-accuracy digital micrometer (iGaging, San Clemente, CA, USA; precision 0.001 mm). Ten measurements (n = 10) were performed from transversal cuts of the films.

### 2.5. Physicochemical Characterization of Films

The FT-IR spectra of films made with pure chitosan and hybrid films containing TvEO were acquired in a Bruker Vertex 70 (Billerica, MA, USA) in the range of 400–4000 cm^−1^ (n = 3). Scanning microscopic approaches (SEM) of films were determined in a JEOL JSM-6610 (Akishima, Kanto, Japan) as previously reported by Morales-Rabanales et al. [11]. Views of the surface and the transversal cut were obtained in triplicate (n = 3). Transmittance and opacity were obtained in accordance with the protocols described by the same authors. Optical analyses were done in triplicate (n = 3).

### 2.6. In Situ Fungistatic Activity of TvEO Hybrid Films

The surface of square films (1 cm^2^) was directly inoculated with 100 conidia from *C. rosea* whereas the surfaces of PDA squares (1 cm^2^) were used to evaluate conidial germination as viability control (n = 10). The conditions of incubation, photoperiod, and verification of fugal proliferation were those reported by Morales-Rabanales et al. [11]. Fresh Hass avocados were disinfected with 40% sodium hypochlorite for 15 min and washed with sanitized distilled water under aseptic conditions (laminar flow cabinet). Subsequently, 1000 conidia from *C. rosea* (determined by Neubauer chamber counting) were injected into the pericarp of avocadoes using a micropipette (200 µL capacity). Inoculated avocados (n = 100) were divided into five groups of 25 samples and covered with the films Ch, FT1, FT2, FT3, and FT4. Healthy uncoated avocados were considered asymptomatic controls. The fruits were incubated in humidity chambers and symptom emergence such as loss of turgor and/or mycelial proliferation was followed for 21 d.

### 2.7. Determination of Fruit Firmness

Fruit firmness was determined using a NEWTRY GY-3 sclerometer (Huizhou, Cantón, China). The hardness tests were done using a pressure head with an 8 mm diameter with a traveled distance of 10 mm. These assays were conducted in treated and untreated avocadoes. The force was reported in kg/cm^2^. This parameter was measured several times in different parts of the fruits (n = 10) [11].

### 2.8. Basic Proximate Analyses and Fatty Acid Determination

The contents of protein, fiber, fat, and reducing sugars were obtained using the methods 920.23, 962.09, 920.39, and 945.66 of the AOAC [13]. The levels of palmitic acid (16:0), palmitoleic acid (16:1), oleic acid (18:1 n–9), and linoleic acid (18:2 n–6) were determined by GC-MS using fatty acid methyl esters prepared from total lipids and authentic standards from Sigma-Aldrich Co. (St. Louis, MO, USA) [14]. An Agilent 5977B GC/MSD chromatograph (Santa Clara, CA, USA) equipped with an HP5-ms capillary column (Palo Alto, CA, USA) was used to determine fatty acid profiling. Running conditions were those reported by Amado et al. [14].

### 2.9. Statistical Analysis

Analysis of variance and Tukey tests (*p* < 0.01) were applied to all treatments. This approach was determined using Statgraphics Centurion ver. XVIII (The Plains, VA, USA).

## 3. Results and Discussion

### 3.1. TvEO Composition and In Vitro Antifungal Activity

Based on a recent investigation [2], C. rosea strain C08-9 was tagged as a causal agent of avocado soft rot in crops from Puebla-Mexico. The same species has been associated with the rot of different crops around the world [15,16]. Thus, new antifungal agents need to be explored to control this new threat to avocado producers. Recent evidence states that essential oils may be a natural alternative for the control of postharvest fruit diseases [10].

The essential oil composition of TvEO used in this investigation revealed thymol as the main volatile (~44%) followed by o-cymene (~16%) and gamma-terpineol (~12.4%). In addition, another 14 volatiles were also found as active constituents of TvEO (Table 1).

The identity of these volatiles was corroborated by comparison with the retention index and by the co-injection of authentic standards (Table 1). The chemical profile of *T. vulgaris* obtained in this investigation was similar to that reported by Borugă et al. [16] who reported p-cymene (8.41%), γ-terpinene (30.90%), and thymol (47.59%) as the most abundant volatiles in *T. vulgaris* grown in Romania. As is known, the essential oil from *T. vulgaris* exerts a strong antimicrobial activity in several foodborne pathogens but little is known about its possible application in the control of seed-borne diseases [17].

Divband et al. [17] have shown that the essential oil of *T. vulgaris,* containing 32.67% thymol and 16.68% p-cymene, exerted minimum inhibitory concentrations (MIC) of 5–20 μg mL^−1^ on native strains of *Fusarium oxysporum*. These authors also reported that the essential oil exerted a downregulation of the Tri4 gene expression in *Fusarium oxysporum* suggesting that this chemical fraction could be used as a natural alternative for the control of seed-borne diseases triggered by *Fusarium* spp. Mohammad and Mehdizadeh [18] reported that the essential oil from *T. vulgaris* exerted strong antifungal activity in *Drechslera spicifera*, *Fusarium oxysporum* f.sp. *cicero* and *Macrophomina phaseolina* which are considered common fungi associated with wilt and rot variants in agronomic crops.

**Figure 1:**
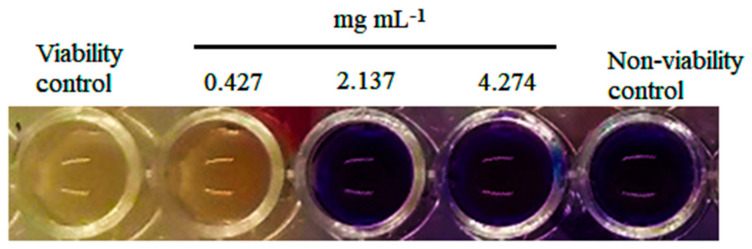
Minimum inhibitory concentration for TvEO on hyphal discs from *Clonostachys rosea* obtained by the broth microdilution method using resazurin as an indicator of cell viability.

The results of the broth microdilution using resazurin revealed that TvEO produced an MIC of 2.13 mg mL^−1^ in *C. rosea* (Figure 1). These assays demonstrated that the essential oil of *T. vulgaris* exerts a fungicide activity because of its ability to stop fungal redox metabolism [12]. The mode of action of thymol in fungal species has revealed its involvement as an irreversible inhibitor of ergosterol biosynthesis [19]. As a consequence, the permeability of the cell membrane increases, inducing cell death. The toxic activity of o-cymene on filamentous fungi is still controversial, however, some studies suggest that this monoterpene may exert a differential fungistatic effect depending on the assayed species [20].

### 3.2. In Situ Fungistatic Activity of Hybrid Films Containing TvEO

**Figure 2:**
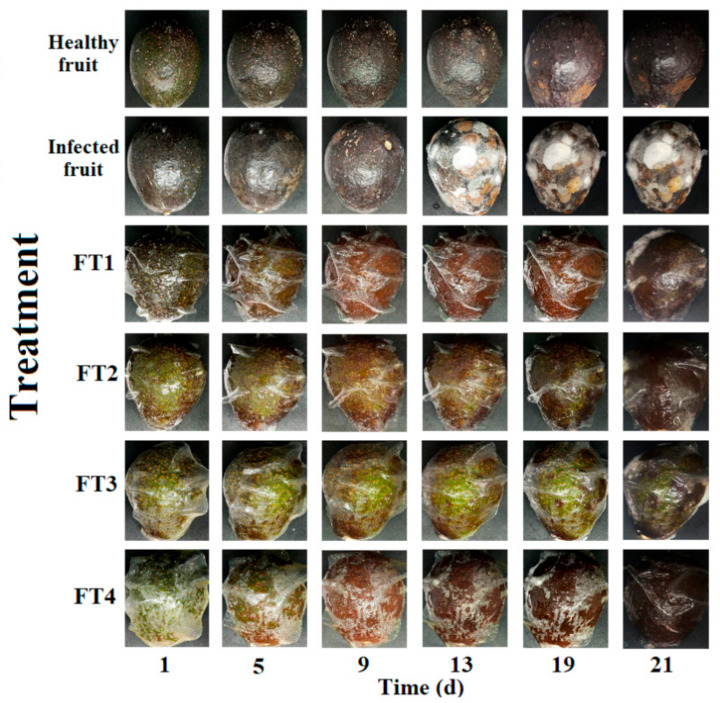
Qualitative effect of the hybrid films FT1–FT4 on soft rot symptom emergence of in avocadoes triggered by *Clonostachys rosea*.

**Figure 3:**
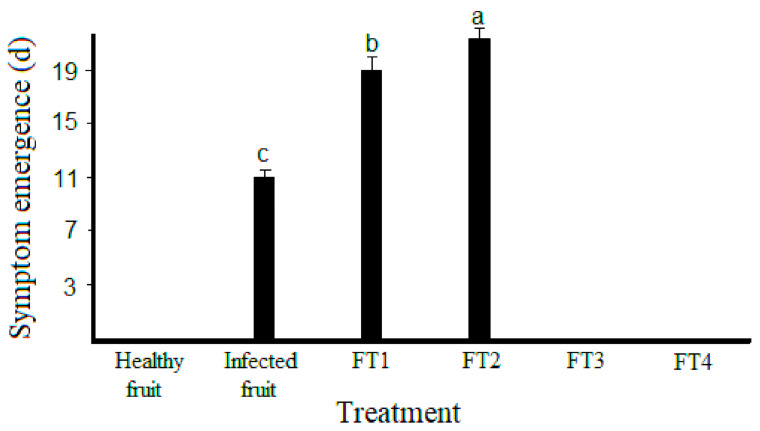
Quantitative delaying effect of the hybrid films FT1–FT4 on the emergence of soft rot symptom in avocadoes triggered by *Clonostachys rosea*. Bars with different letters indicate means with statistically significant differences (*p* < 0.01). Avocadoes treated with the films FT3 and FT4 did not show rot symptoms after 21 d post-inoculation.

The four hybrid coatings generated in this work (FT1–FT4) exerted a differential fungistatic effect in avocadoes previously inoculated with viable conidia from *C. rosea* (Figure 2). Interestingly, a directly proportional relationship between in situ fungistatic activity and the concentration of TvEO in the films was observed. According to our results, infected fruits produced rot symptoms from the ninth-day post-inoculation, however, the films FT1 and FT2 retarded the appearance of symptoms until the eleventh and nineteenth days post-inoculation, respectively (Figure 3).

Statistically significant differences (*p* < 0.01) were observed among infected fruits and those treated with the films FT1 and FT2 (Figure 3). Remarkably, the fruits treated with the films FT3 and FT4 did not show any symptoms of rot (Figure 2 and Figure 3). This effect was observed until the thirtieth day, when the experiment was concluded (data not shown). This evidence suggested that the hybrid films containing 1% and 1.3%TvEO were the most effective to retard the soft rot in Hass avocadoes caused by *C. rosea*. Unexpectedly, the films FT3 and FT4 retarded the black pigmentation of avocadoes suggesting that the volatiles of TvEO avoid the natural production of melanin (Figure 2). Nevertheless, further studies focused on the determination of melanin content should be performed to endorse this observation.

According to Pacheco-Hernández et al. [21], the essential oil from *Lepidium virginicum* exerted a protective effect on the conservation of carotenoids and anthocyanins of tamarillo fruit. These compounds confer the characteristic color of the pulp and pericarp of this exotic fruit [20]. A similar protective effect was reported by Pérez-Vázquez et al. [10] on the conservation of the carotenoids of manzano pepper. On the other hand, the antifungal activity of hybrid films containing chitosan and the essential oil from *Schinus molle* demonstrated its effectiveness on cherry tomatoes infected with *Fusarium oxysporum* [11]. Since the components used to create the films FT1–FT4 are considered natural fibers and condiments, their consumption seems to be safe for human health [11].

### 3.3. Effect of Hybrid Films Containing TvEO on Fruit Firmness

**Figure 4:**
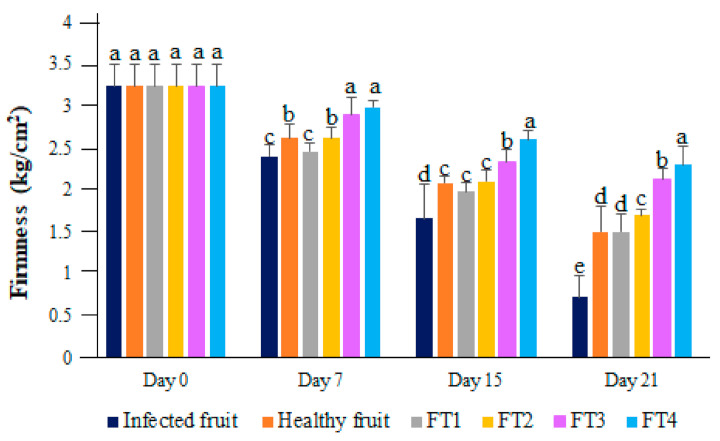
Firmness of avocadoes treated with the films FT1–FT4 after inoculation with *Clonostachys rosea*. Bars with different letters indicate means with statistically significant differences by ANOVA-Tukey test (*p* < 0.01).

The firmness of avocado fruits was dramatically affected by the infection of C. rosea from the fifteenth day to the twenty-first day post inoculation (Figure 4). Interestingly, the protective effect of the films FT3 and FT4 was observed from the seventh day to the twenty-first day post inoculation. According to our results, the firmness of avocadoes treated with the films FT3 and FT4 was better conserved (*p* < 0.01) than that of healthy fruits (Figure 4). Similar results were observed for hybrid films containing high concentrations of the essential oil from *Schinus molle* which exerted a protective effect on cherry tomatoes infected with *Fusarium oxysporum* [11]. Comparable results have been observed for chitosan films saturated with different essential oils as edible coatings of papayas and mangoes [22]. Our results suggest that the hybrid films generated in this investigation promote the conservation of this important parameter for fruit quality.

### 3.4. Effect of Hybrid Films on the Nutritional Content of Hass Avocadoes

**Figure 5:**
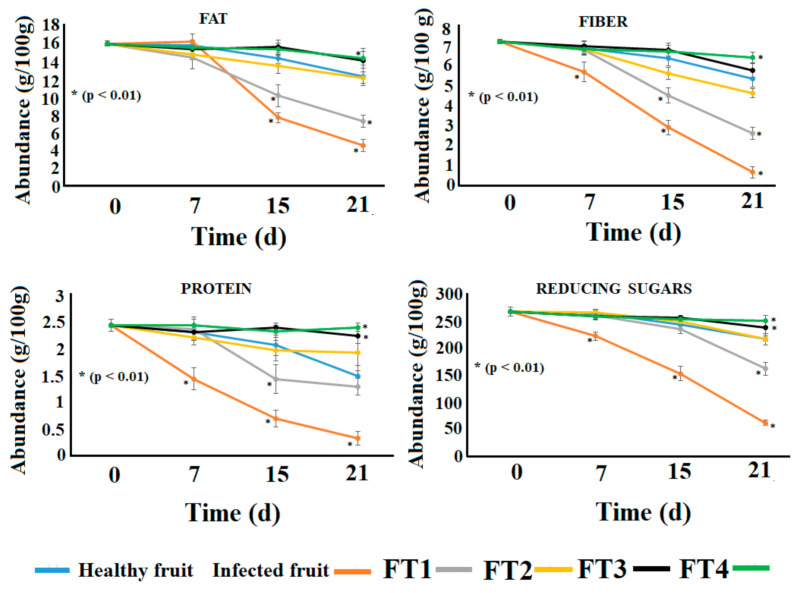
Nutritional parameters of Hass avocadoes treated with the films FT1–FT4 after inoculation with *Clonostachys rosea*. Points with asterisks indicate means with statistically significant differences by ANOVA-Tukey test in comparison with healthy fruits (*p* < 0.01).

According to our observations, the nutritional content of the avocadoes infected with *C. rosea* dramatically decreased on the fifteenth day post inoculation (Figure 5). Nevertheless, the levels of fiber, reducing sugars, and protein started to decrease on the seventh day post inoculation. Interestingly, the reduction of the fiber level was coincident with that of firmness on the seventh day post inoculation (Figure 4 and Figure 5). As is known, the amount of fiber is directly related to the firmness degree [23]. Thus, the effect of both parameters should be related to the parasitic activity of *C. rosea*. On the other hand, the avocadoes treated with the films showed differential conservation of the nutritional parameters (Figure 5). Though all the films exerted a protective effect on the nutritional content of avocadoes, the most effective films in avoiding degradation of fiber, fat, protein, and reducing sugars were those containing the highest concentration of essential oil (FT3 and FT4). These films prevented around 70% degradation of the nutritional components.

Remarkably, the levels of the four parameters decreased in healthy fruits on the twenty-first day post inoculation, probably as a consequence of the natural ripening of avocado fruit [24]. Interestingly, the avocadoes inoculated with *C. rosea* showed better conservation of these parameters compared with healthy uncoated fruits at the twenty-first day.

### 3.5. Effect of Hybrid Films on the Nutraceutical Content of Hass Avocadoes

**Figure 6:**
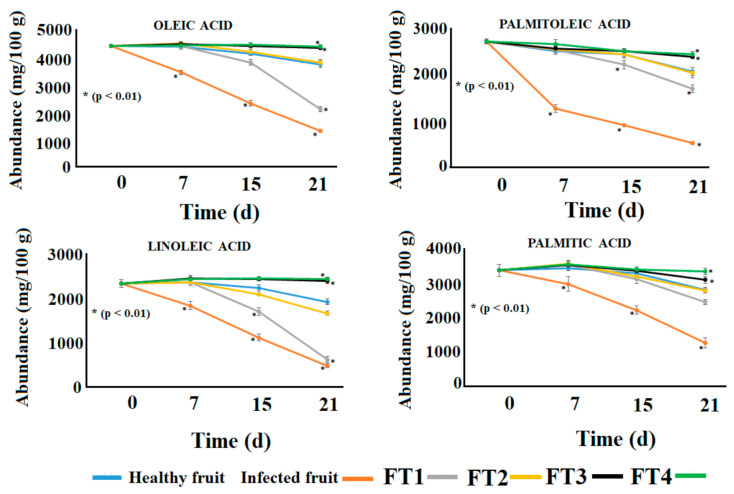
Fatty acid content of avocadoes treated with the films FT1–FT4 after inoculation with *Clonostachys rosea*. Points with asterisks indicate means with statistically significant differences by ANOVA-Tukey test in comparison with healthy fruits (*p* < 0.01).

The fatty acid content of avocadoes is the most relevant nutraceutical parameter. Avocado oil is considered a source of monounsaturated fatty acids (MUFA) which improve human health and mask the taste and texture of the dietary fiber [25]. The consumption of MUFA has been associated with substantial increases in the levels of HDL-cholesterol preventing metabolic syndrome [25]. The Hass avocado, harvested in the northern highlands of Puebla-México, contained substantial levels of oleic acid (~5 g 100 g^−1^), palmitoleic acid (~3 g 100 g^−1^), linoleic acid (~2.5 g 100 g^−1^), and palmitic acid (~3.5 g 100 g^−1^) (Figure 6). An evident decrease in the levels of these compounds was observed in diseased avocadoes on the seventh day post inoculation (Figure 6). As observed for basic nutritional parameters, the fatty acid content of Hass avocadoes was conserved by the application of the hybrid films containing TvEO. However, the most effective coatings were FT3 and FT4 (Figure 6).

### 3.6. Physicochemical Characteristics of TvEO Hybrid Films

The hybrid films FT1, FT2, FT3, and FT4 showed similar consistency, but slight color differences were observed as the concentration of essential oil increased. However, these films showed minor color differences if compared with those made with pure chitosan (Ch).

**Figure 7:**
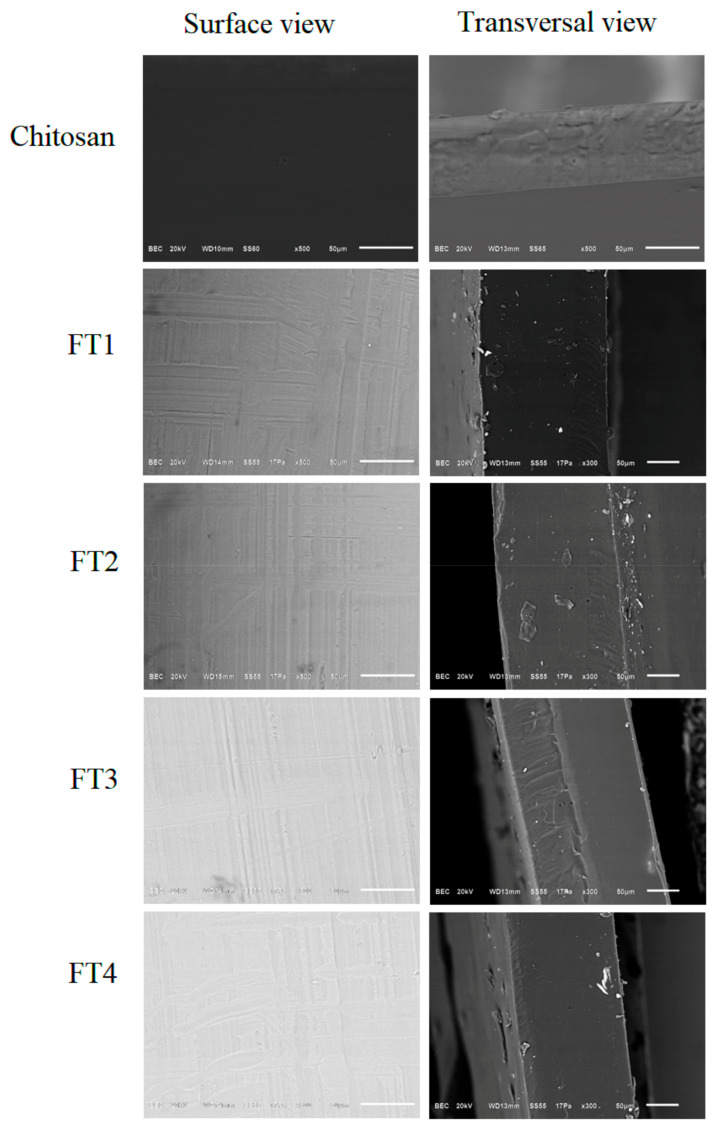
SEM micrographs of the surface and the inner part of the hybrid films FT1–FT4 containing different concentrations of TvEO (0.4–1.3%). The features of films made with pure chitosan are included and the scale bar is equivalent to 50 µM.

The homogenous texture of the film’s surface was corroborated by SEM approaches and interestingly, the transversal cut revealed an acceptable homogeneity of the inner part of all films studied (Figure 7). These results strongly suggest that TvEO has good miscibility in chitosan at least at the concentration assayed [11]. Other works focused on the design of hybrid films reported the presence of irregularities or inner cavities which are produced by the low miscibility of the essential oil in chitosan [11]. Interestingly, the thickness of the films Ch, FT1, FT2, FT3, and FT4 was 0.052, 0.182, 0.238, 0.259, and 0.302 mm, respectively. These results indicate that the highest concentrations of TvEO increased the thickness of the studied films.

**Figure 8:**
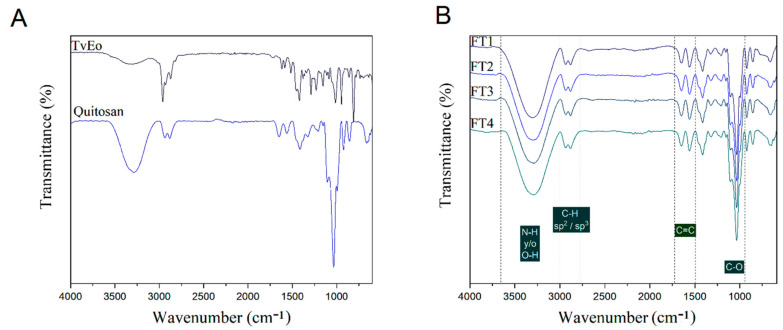
FT-IR spectra of TvEO and films made with pure chitosan (**A**) and comparative stacking of the four hybrid films containing different concentrations of TvEO (**B**).

Clear differences were observed in the FT-IR spectrum of TvEO and films made with pure chitosan (Figure 8A). However, no differences in the FT-IR profile were observed among chitosan films containing different concentrations of TvEO (Figure 8B). 

The FT-IR spectrum of the thyme essential oil (Figure 8A) showed a signal at 3329 cm^−1^ due to the stretching of the O–H which was strongly linked to the phenol group of thymol. Damián et al. [26] have reported the presence of band widening, which is simultaneously related both to the formation of hydrogen bonds and with a decrease in the absorption frequency (3000 cm^−1^–3600 cm^−1^). These observations may be related to the interaction of hydroxylated volatiles with polar groups of chitosan. The signal observed at 2930 cm^−1^ represents the typical stretching of the asymmetric and symmetric vibrations of the CH_2_ group [27]. The peaks observed at 2962 cm^−1^ and 2877 cm^−1^ were related to the stretching vibration of the CH_3_ which could be associated with the presence of beta-cubebene. On the other hand, the signal observed at 1718 cm^−1^ indicates the bending of C-H, and this stretching vibration would correspond to the carbonyl group from (2*E*)-Hexenyl acetate. The peak observed at 1624 cm^−1^ was associated with the stretching of C=C–C. The presence of an aromatic ring is observed at 1585 cm^−1^ which indicates the main functional group of thymol. The signal detected at 1459 cm^−1^ was associated with the bending of the CH_2_ group of alpha-pinene [26]. The presence of this compound was endorsed by the signal detected at 1016 cm^−1^. The peaks observed at 1484–1359 cm^−1^ coincided with the bending vibration of the group C–H from camphene [28]. The signal detected at 1424 cm^−1^ suggested the asymmetric bending of -CH3 whereas the signals detected at 1285 and 1226 cm^−1^ were associated with the stretching of C–O–C. The signal detected at 940 cm^−1^ has been related to the presence of the gamma-terpinene which is one of the major volatiles of TvEO [27]. The signal detected at 806 cm^−1^ corresponded to the overlay of C–H groups included in thymol and o-cymene.

The FT-IR spectrum of pure chitosan films showed a typical signal at 3282.09 cm^−1^ which is related to the stretching vibration of O-H and NH_2_ (Figure 8A). The signals detected at 2937.01 and 2883.66 cm^−1^ were related to the CH_3_ stretching of chitosan. In this context, the signals observed at 1414.94 cm^−1^ and 1322.35 cm^−1^ indicate the symmetric (δs) and asymmetric (δas) stretching of CH_3_. Morales-Rabanales et al. [11] have stated that these signals could be related to the presence of electrostatic bonds produced between the polar groups of the compounds dissolved in the essential oil and those from chitosan.

**Figure 9:**
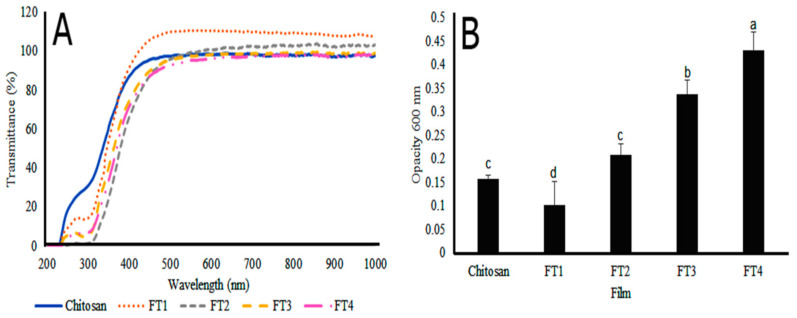
Transmittance (**A**) and opacity (**B**) of films made with pure chitosan and the hybrid films FT1-FT4 containing different concentrations of TvEO. Bars with diverse letters indicate statistically significant differences (*p* < 0.01) by ANOVA-Tukey test.

A desirable property of a food package is related to its capacity to avoid the penetration of UV radiation [29]. The results of transmittance reveal that UV light (190–300 nm), and visible light (350–800 nm) penetration decreased in the films FT3–FT4 (Figure 9A). These films were more opaque than the films FT1 and FT2 because of their TvEO concentration (Figure 9B). Interestingly, the film FT1 had similar optical properties as those of the films made with pure chitosan (Ch) at 700–900 nm. These results reveal that the films containing the highest amount of TvEO exert better protection against UV light than those containing low concentrations of TvEO. Thus, the films FT3 and FT4 would prevent possible damage and premature ripening of avocadoes [30].

**Figure 10:**
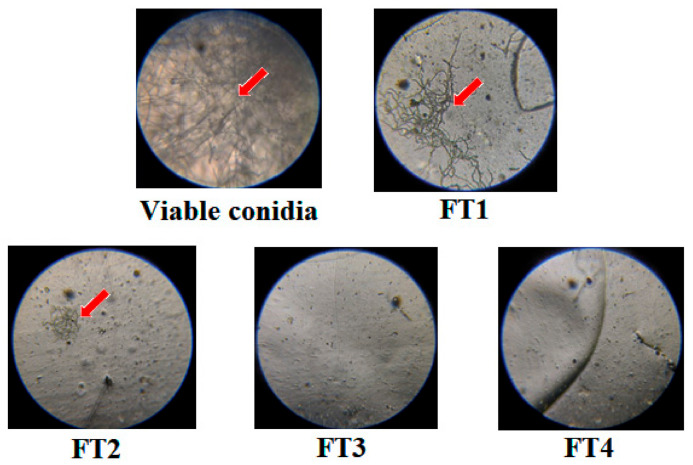
Germination of viable conidia from *Clonostachys rosea* on the surface of the films FT1–FT4. The control of germination was performed on PDA (viable conidia). Red arrows indicate the proliferation of mycelium.

The results of the conidial germination on films surface demonstrate that all films exerted a differential fungistatic effect on the proliferation of *C. rosea*. Nevertheless, this effect was proportional to the concentration of TvEO added (Figure 10). As observed in Figure 10, conidial emergence and mycelium proliferation were only noted on the surface of the films FT1 and FT2. However, the proliferation of mycelium in these films was dramatically reduced in comparison with that of viable conidia growing in PDA. This evidence endorses the idea that the films FT3 and FT4 were the best coatings to prevent the in situ proliferation of *C. rosea* and may be useful to avoid the germination of viable conidia spread by air. Other composites described by Ganeson et al. [31] which were made with gelatin and thyme essential oil, have shown interesting results in the conservation of fruit quality. However, these composites were designed with the aim to improve the shelf life of healthy apples instead of diseased apples.

Unlike the latter investigation, the present investigation was focused on extending the shelf life of infected avocadoes instead of healthy fruits. To the best of our knowledge, few investigations have been performed on the antifungal in situ effects of hybrid films containing essential oils in avocadoes. Nevertheless, Chávez-Magdaleno et al. [32] performed similar in vitro experiments to those of our investigation. However, these authors measured the radial growth of wild strains of *Colletotrichum* sp. isolated from diseased avocadoes on the surface of chitosan films combined with eucalyptus and cinnamon essential oils. A closely related work reported by Correa-Pacheco et al. [33] stated the effect on nanostructured coatings containing thyme essential against the in situ growth of *Colletotrichum gloeosporioides* in avocadoes. These authors reported 60% antifungal efficiency of these coatings retarding the emergence of anthracnose symptoms up to eight days. According to our results, the films FT3 and FT4 generated in the present investigation showed 100% efficiency in delaying rot symptom emergence caused by *C. rosea* for at least 21 days. Coincidently, both investigations coincide in the fact that the use of chitosan coatings with thyme essential oil improved fruit firmness in comparison with untreated fruits.

## 4. Conclusions

The hybrid films FT1–FT4 containing chitosan and TvEO showed a differential in situ fungistatic effect on *C. rosea*, an emergent postharvest phytopathogen of avocado fruit. The films FT3 and FT4 showed the best fungistatic properties as well as desirable properties of a food package such as low transmittance and opacity. The miscibility of TvEO in chitosan was demonstrated by the homogeneity of the film’s surface as well as by the inspection of the inner part of the film. The new coatings developed in this investigation retarded the emergence of rot symptoms for at least 21 days after inoculation with viable conidia from *C. rosea*. The application of these coatings was translated into the conservation of the nutritional and nutraceutical parameters of avocadoes previously infected with the phytopathogen. Remarkably, these parameters were better conserved in avocadoes treated with hybrid films than in uncoated healthy fruits.

## Figures and Tables

**Table 1:** Chemical profile of the leaf essential oil from *Thymus vulgaris* obtained in Puebla-México (TvEO).

Compound	Retention Index	Abundance (%)
Alpha-Pinene ^1^	939	2.5
Camphene	946	2.8
Beta-Pinene ^1^	974	2.1
Alpha-Phellandrene	1002	0.5
(2E)-Hexenyl acetate	1010	2.9
o-Cymene ^1^	1022	15.7
Gamma-Terpinene ^1^	1054	12.4
Linalool ^1^	1095	1.6
1-Terpineol	1130	2.4
Camphor ^1^	1141	0.5
Borneol ^1^	1165	1.4
Alpha-Terpineol	1186	0.3
Thymol, methyl ether	1232	1.7
Thymol ^1^	1289	43.6
Beta-Cubebene	1387	3.3
Beta-Elemene	1389	0.5
Beta-Caryophyllene ^1^	1417	1.2
Total		95.4

^1^ Identity corroborated by co-injection of authentic standards.

## Data Availability

Not applicable.
